# EEG-Based Computer Aided Diagnosis of Autism Spectrum Disorder Using Wavelet, Entropy, and ANN

**DOI:** 10.1155/2017/9816591

**Published:** 2017-04-18

**Authors:** Ridha Djemal, Khalil AlSharabi, Sutrisno Ibrahim, Abdullah Alsuwailem

**Affiliations:** Electrical Engineering Department, College of Engineering, King Saud University, Box 800, Riyadh 11421, Saudi Arabia

## Abstract

Autism spectrum disorder (ASD) is a type of neurodevelopmental disorder with core impairments in the social relationships, communication, imagination, or flexibility of thought and restricted repertoire of activity and interest. In this work, a new computer aided diagnosis (CAD) of autism ‎based on electroencephalography (EEG) signal analysis is investigated. The proposed method is based on discrete wavelet transform (DWT), entropy (En), and artificial neural network (ANN). DWT is used to decompose EEG signals into approximation and details coefficients to obtain EEG subbands. The feature vector is constructed by computing Shannon entropy values from each EEG subband. ANN classifies the corresponding EEG signal into normal or autistic based on the extracted features. The experimental results show the effectiveness of the proposed method for assisting autism diagnosis. A receiver operating characteristic (ROC) curve metric is used to quantify the performance of the proposed method. The proposed method obtained promising results tested using real dataset provided by King Abdulaziz Hospital, Jeddah, Saudi Arabia.

## 1. Introduction 

*Autism spectrum disorder* (ASD) is a neurodevelopment disorder that includes (*classic*)* autism*,* Asperger's syndrome*, and* pervasive developmental disorder not otherwise specified* (PDD-NOS) [1]. ASD diagnosis is mainly based on behavioral and interview test such as using diagnostic and statistical manual of mental disorders, 5th edition (DSM-5) [2]. Different types of autism were previously classified as different disorders, but now in DSM-5 all fall under one umbrella that is ASD. Computer aided diagnosis (CAD) system is a computer system (or program) built to aid clinician or medical doctor to diagnose certain disease or disorder. CAD gives second opinion for the clinician to diagnose the disorder. CAD system is not intended to diagnose by itself but as an assisting tool for clinician for diagnosing therefore saving the time and increasing the accuracy.

Recently, researchers tried to develop computer aided autism spectrum disorders diagnosis based on* electroencephalogram* (EEG) signals [3]. EEG has high temporal resolution and is relatively cheap and widely available for clinicians. Applying Fourier transform directly to such signal is not practically suitable because the nature of EEG signals is rather complex, nonlinear, and nonstationary. Wavelet transform is able to represent the EEG signal in multiscale time-frequency domain and captures subtle changes in the signal. This research work aims to investigate a new autism diagnosis procedure based ‎on* discrete wavelet transform* (DWT) combined with Shannon entropy and* artificial neural network* (ANN).

DWT decomposes the EEG segment into several frequency subbands. Several statistical features (mean, variance, and standard deviation) and several entropy functions (log energy entropy, threshold entropy, Renyi entropy, and Shannon entropy) are used to extract the feature from each EEG subband. Then ANN classifies the corresponding EEG segment based on these extracted features. The best classification accuracy is obtained using Shannon entropy as features extraction. The rest of this paper is organized as follows: ‎Section 2 provides brief literature review to the topic.  Section 3 highlights the EEG dataset used in this work and the feature extraction and ‎the classification methods. Experimental results are presented and discussed in Section 4.  Section 5 concludes the paper and highlights the future research direction.‎

## 2. Literature Review 

The development of automatic mechanism to ‎analyze brain signals would improve the ‎speed and the accuracy of the clinician to diagnose certain disease or disorder. Several computer aided diagnosis (CAD) methods for autism diagnosis have been investigated‎ by several previous studies. In the work presented by Sheikhani et al. [4], the datasets were recorded by 21 electrodes with both earlobes chosen as common referential electrodes and extracted from two groups: 10 (9 boys and 1 girl) ASD and 7 (4 boys and 3 girls) non-ASD children. A short time Fourier transform (STFT) technique was used to extract EEG signal features and then applied as an input to nearest neighbors (KNN) classifier to get classification accuracy up to 82.4%. In their later paper [5], the authors improved the method and used larger data for testing (17 ASD and 11 normal subjects) which obtained up to 96.4% distinction level.

Ahmadlou et al. [6] investigated fractal dimension (FD) to measure complexity and dynamical changes in ASD brain. The method was tested on a database of eyes-closed EEG data obtained from two groups: 9 ASD and 8 non-ASD children. The dataset was recorded according to 10–20 international system, each consisting of 19 channels, and digitized with sampling rate of 256 Hz. An accuracy of 90% was achieved with a radial basis function classifier. Later, the same group also presented ASD diagnosis using visibility graph (VG) [7] and fuzzy synchronization likelihood (fuzzy SL) and enhanced probabilistic neural network (EPNN) classifier [8]; the two proposed methods obtain around 95.5% accuracy.

Fan et al. 2015 [11] presented spectral features of EEG signals from a 14-channel EEG neuroheadset, together with therapist ratings of behavioral engagement, enjoyment, frustration, boredom, and difficulty to train a group of classification models. They used seven classification techniques and compared the results: Bayes network, naive Bayes, support vector machine (SVM), multilayer perceptron, *K*-nearest neighbors (KNN), random forest, and decision tree classifier (J48), to obtain the classification accuracy ranging 75–85%.

It was reported by Bosl et al. [9] that an EEG dataset was collected from 79 subjects: 46 ASD and 33 non-ASD subjects. The EEG dataset was recorded by 64-channel Sensor Net System and Net Station software, amplified, band-pass filtered at 0.1 to 100.0 Hz, and sampled at a frequency of 250 Hz. They used minimum mean square error (MMSE) as a feature vector and then the multiclass *k*-nearest neighbors (KNN), the support vector machine (SVM), and naive Bayesian (NB) classification algorithms have been applied to classify typical signal and autistic signal. The classification accuracy is over 80% at age of 9 months. Classification accuracy for boys was close to 100% at age of 9 months and ranged between 70% and 90% at 12 and 18 months. For girls, classification accuracy was highest at age of 6 months but declines thereafter.

In Alhaddad et al. [10] the dataset was collected from 12 children: 8 (5 boys and 3 girls) with ASD and 4 (all of them are boys) with non-ASD. The dataset was recorded by g.tec EEG acquisition system which has 16 channels with AFz electrode as GND and right ear lobe as reference and then filtered using band-pass filter with a frequency band (0.1–60 Hz) and digitized at 256 Hz. The notch filter was also used at 60 Hz. Optimum preprocessing techniques were used in this study. They used two feature extraction techniques: time and frequency domains (raw data and FFT). Fisher linear discriminant (FLD) is used as classifier. They obtained a classification accuracy up to 90%. Later Alsaggaf and Kamel [12] used the same dataset and processing techniques used by Alhaddad for autism disorders diagnosis and obtained 80.27% accuracy.

## 3. Methods and Materials

### 3.1. Methods Overview

**Figure 1:**
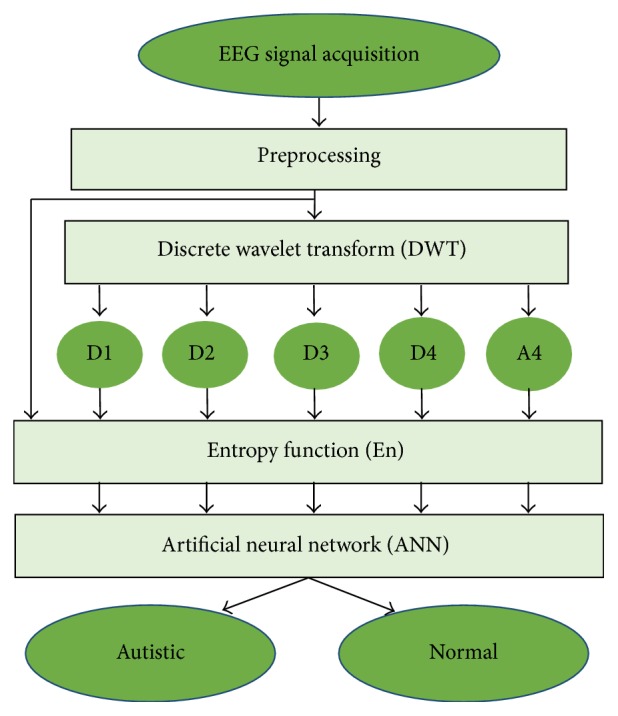
Block diagram of the proposed method.

Figure 1 shows the overview of the proposed method. In the beginning, some preprocessing is done in the input EEG. This preprocessing step includes segmentation process, filtering, and overlapping the EEG segment.

After preprocessing, EEG segment as an input is fed to discrete wavelet transform (DWT). DWT dismantles the EEG segment into detail coefficients (D1–D4) and the corresponding approximate coefficient (A4) for EEG subbands such as delta, theta, alpha, beta, and gamma. Entropy values are then extracted from the original EEG segment and these coefficients to estimate the time series distribution and to reduce the dimension of the extracted features. Several statistical features (mean, standard deviation, etc.) are used also for feature extraction. Artificial neural network (ANN) is used as classifier.

### 3.2. Dataset

**Figure 2:**
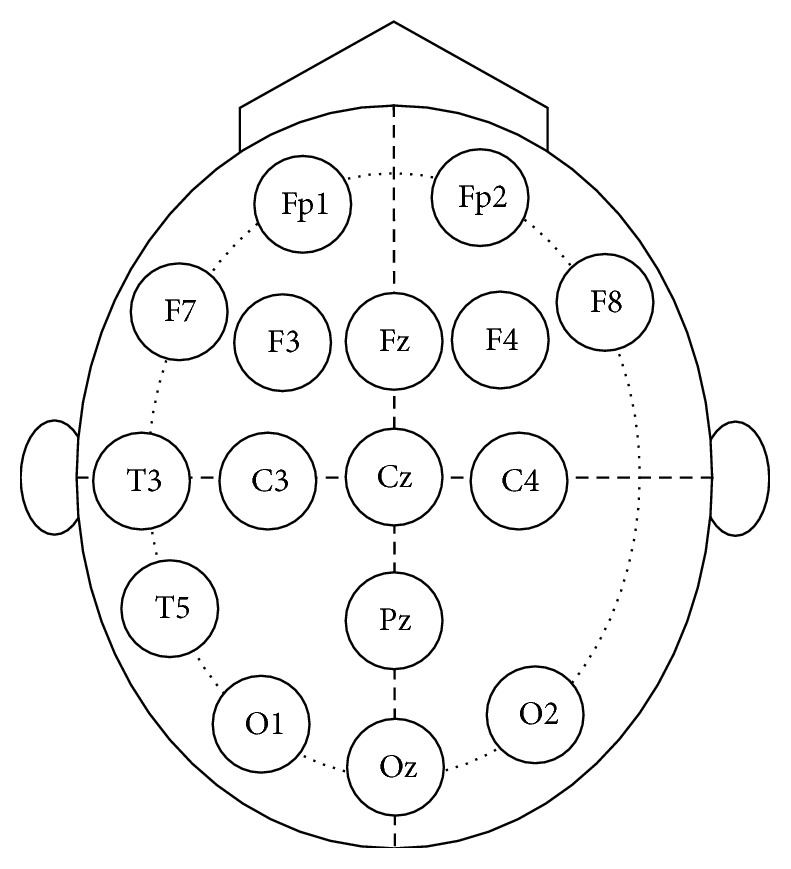
Electrodes placement of autism data acquisition system.

Autism dataset used in this work is provided by King Abdulaziz University (KAU) Brain Computer Interface (BCI) Group, Jeddah, Saudi Arabia (see http://malhaddad.kau.edu.sa/Pages-BCI-Datasets.aspx). The data recording was done in the laboratory of KAU Hospital. We obtained permission to use the data from our college in KAU. To ensure the anonymity of the subjects, all personal information is not published (name, address, etc.). The data is described in more detail in [10]. Dataset was recorded in relaxing state and divided into two groups: the first one is called normal group and contains ten healthy volunteer subjects (all of them are males, age 9–16 years) with normal intelligence and without any mental disorder. The second one is called autistic group and contains nine subjects (six males and three females, age 10–16 years) with autism spectrum disorders. The EEG signals were recorded from subjects scalp in relaxing state by EEG data acquisition system that contains the following components: a g.tec EEG cap with high accuracy Ag/AgCl sensors (electrodes), g.tec USB amplifiers, and BCI2000 software. The data acquisition system has 16 channels, which are labeled based on 10–20 international acquisition system as shown in Figure 2. All electrodes, 16 channels, are used to record the EEG data.

**Figure 3:**
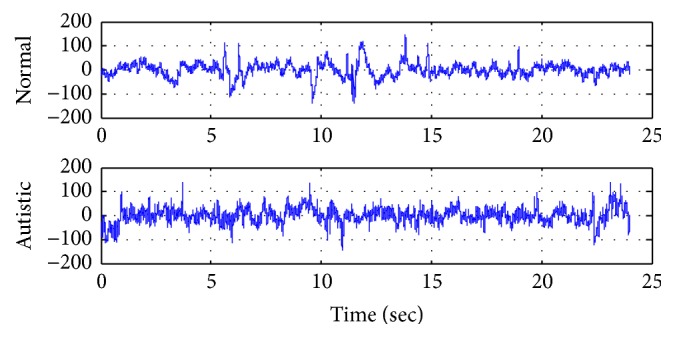
Example of raw normal and autistic EEG signal.

The dataset was filtered by band-pass filter with pass band frequency (0.1–60 Hz) and notch filter with stop band frequency (60 Hz) and all EEG signals were digitized at frequency sampling 256 Hz. The EEG recording varies from around 12 to 40 minutes for autistic subjects with a total up to 173 minutes, while, for normal subjects, recording varies from 5 to 27 minutes with a total up to 148 minutes.  Figure 3 shows ‎the typical EEG signals for normal and autistic subjects. For more detailed information about the dataset, please refer to Alhaddad et al. [10].

### 3.3. Preprocessing

In the preprocessing stage, the acquired EEG signal will be treated through a signal processing block to remove the artifacts and noises in the signal. EEG signals are usually burred with noises derived from many factors such as bad electrode location and dirty hairy leather [13]. Furthermore, the presence of these artifacts is also due to the interference with signals coming from other parts of the body such as heart and muscle activities. It is mandatory to remove all artifacts and enhance the signal to noise ratio by filtering the acquired data. The filtering block aims to remove artifacts, improve the stationary, and increase accuracy. Many alternatives have been explored in [14] as follows:

- The first one is using frequency domain transforms such as fast Fourier transform (FFT) or using time-frequency domain such as discrete wavelet transform (DWT).
- Subtracting artifacts from the acquired signal: this technique requires an average artifacts template estimation to be subtracted from the original EEG signal.
- Using the same static filtering for all subjects like finite impulse response (FIR) and infinite impulse response (IIR) filters: FIR filters like Equiripple and Kaiserwin are based on Parks-McClellan algorithm using the Remez exchange algorithm and Chebyshev approximation theory to design filters with an optimal_t between the desired and the actual frequency responses [15].
- Using adaptive filtering techniques: one of the most important interferences in EEG signals is ocular (or eye) artifact. He et al. [16] used adaptive filtering to cancel ocular artifacts by using electrooculogram (EOG) recording, however, providing EOG recording inconvenient and uncomfortable for the patient. The removal of eye-artifact from EEG signal is also presented in [17] by applying the independent component analysis (ICA) to extract information from electrodes close to eyes. Chan et al. in [18] presented an ocular-artifact removal technique based on adaptive filtering using reference signal from the ocular sources components (SCs), which avoids the need for parallel EOG recordings.

In most of the previous works (such as the work of Sheikhani et al. [4, 5] and also Ahmadlou et al. [6–8]), they used artifact-free data. The EEG data is manually prepared or selected by expert. This scheme is good for research and initial analysis, but not for clinical use. The system should be robust and able to automatically tackle noises and artifacts in the EEG signal by necessary preprocessing and artifacts removal. However, if the preprocessing and artifacts removal are not designed properly, they might remove also the useful information in the EEG leading to inconsistent accuracy values.

Based on the above-mentioned preprocessing design exploration, we used independent component analysis (ICA) for eye-artifact removal and elliptic band-pass filter for filtering. We follow [17] that employed ICA and adaptive filtering to remove ocular-artifacts. Electrodes closed to eye (FP1, FP2, F7, and F8) are used as reference signals for ocular-artifacts removal. After ocular-artifact removal, the signals are then filtered using elliptic band-pass filter. Elliptic band-pass provides better experimental accuracy compared with other filters like Chebyshev type I and type II and Butterworth which are IIR filters [19]. Furthermore, the implementation of the elliptic filter requires less memory and calculation and provides reduced time delays compared with all other FIR and IIR filtering techniques. The proposed computer aided classification system is required to segment each EEG signal into fixed length windows. In our experimental analysis, each EEG signal was segmented into overlapping and nonoverlapping windows.

### 3.4. Wavelet Decomposition

Wavelet transform techniques are widely used in EEG signal processing for time-frequency decomposition. There are two types of wavelet analysis: continuous wavelet transforms (CWT) and discrete wavelet transforms (DWT). The CWT one is applied for extracting event related potential time-frequency features of the nonstationary EEG signal and combined with the *T*-student algorithm to choose features that are more effective, resulting in significant classification improvement [20]. However, one obvious drawback of the CWT technique is that it requires excessive calculations. Therefore, we used the DWT in the proposed work to decompose a given EEG signal into approximation and detail coefficients to obtain a first level of decomposition.

**Figure 4:**
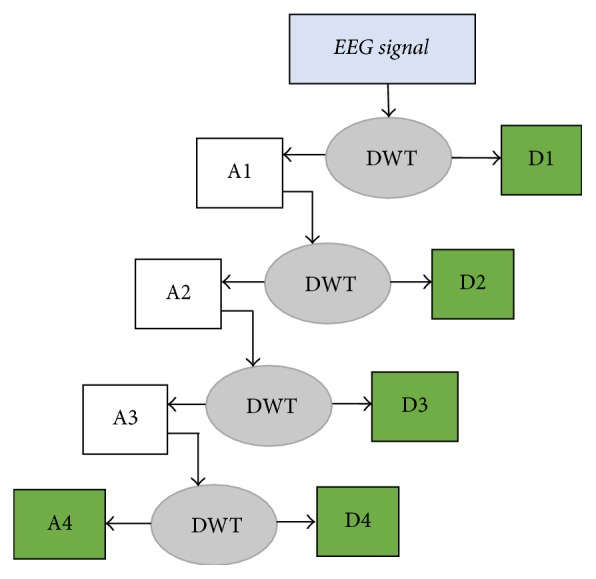
EEG signal decomposition through 4-level DWT.

The approximation coefficients in every level are further decomposed into next level of approximation and detail coefficients as shown in Figure 4. Selection of decomposition levels and type of mother wavelet are very important in analysis of certain signal using DWT. In this work we used 4-level DWT decomposition with* Daubechies-four* (db4) mother wavelet in order to extract five EEG subbands and to achieve better results in features extraction stage. The features are extracted from the detailed coefficients at various levels (D1–D4) and from the approximation coefficients at the last level (A4). Statistical features, such as mean or standard deviation and entropy value, will be calculated from these five wavelet coefficients (D1, D2, D3, D4, and A4) to construct the feature vector.

**Figure 5:**
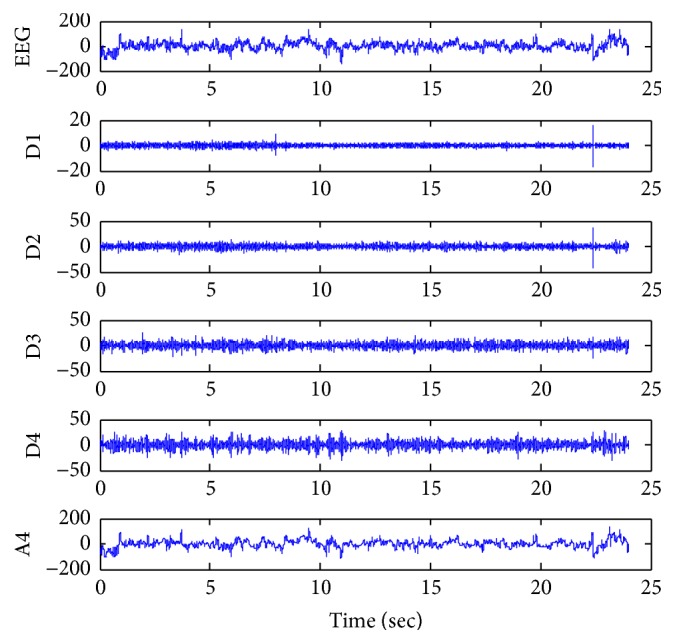
Approximate and details coefficients extracted from an EEG segment of autistic subject.

The frequency bands of EEG signal corresponding to 4-level DWT decomposition with sampling frequency of 256 Hz on the EEG signal are shown in Table 1. As shown in Table 1, the wavelet coefficients are corresponding to several EEG subbands:* delta* (1–4 Hz),* theta* (4–8 Hz),* alpha* (8–15 Hz),* beta* (15–30 Hz), and* gamma *(30–60 Hz). Different frequency subbands can reveal the characteristics of the time series of EEG signal.  Figure 5 shows an example of approximation and details coefficients extracted from an EEG segment of autistic subject.

### 3.5. Feature Extraction

Many features can be extracted from the time series of EEG signal such as using statistical features or nonlinear features (entropy). Several previous studies show the effectiveness of using entropy to analyze EEG signal, such as for epilepsy diagnosis [21, 22] and autism diagnosis [9]. Entropy can be used to ‎measure the complexity, regularity, and the statistic ‎quantification of time series data‎ such as EEG. Bosl et al. [9] have investigated the possibility of using EEG complexity as a biomarker for ASD risk. The abnormal nonlinearity and complexity in the brain signal may reveal brain disorder or cognitive impairments. These motivate us to do further investigation about using entropy as a tool to diagnose ASD.

In this study we investigate five statistical features (mean, standard deviation, variance, skewness, and kurtosis) to be extracted from each DWT output coefficient. There are many types of entropy function. We investigate several entropy functions in this study: log energy and threshold entropies, Renyi entropy, and Shannon entropy. The description for each this entropy is given as follows.

#### 3.5.1. Log Energy Entropy

Log energy entropy is a type of wavelet entropy. We suppose a signal $x=\left[\begin{array}{ccccc} x_{1} & x_{2} & x_{3} & \cdots & x_{n} \end{array}\right]$ probability distribution function denoted by *p*(*x*_*i*_), where *i* is the index of signal elements, and the log energy then entropy is defined as

(1) $$\begin{array}{c} H=\overset{n}{\underset{i=1}{\sum }}\log\left(\;{p_{i}}^{2}\;\right). \end{array}$$

#### 3.5.2. Threshold Entropy

Threshold entropy is a statistical function used to measure the number of times that the discrete wavelet coefficients are larger than the threshold. The threshold selected in this paper is to equal 0.2. This threshold value is selected based on try-and-error to obtain the best accuracy.

#### 3.5.3. Renyi Entropy

Renyi entropy is a statistical function to measure the diversity and randomness of the discrete signal distribution and to estimate uncertainty of the discrete signal. It can be calculated by the following equation:

(2) $$\begin{array}{c} H=\frac{1}{1-\alpha }\log\left(\;\overset{n}{\underset{i=1}{\sum }}p_{i}^{\alpha }\;\right), \end{array}$$

where *α* is the order of Renyi function, *α* ≥ 0 and *α* ≠ 1, and *p* is the probability of the discrete signal variables.

#### 3.5.4. Shannon Entropy

Shannon entropy is a technique used to expect the average value of the information contained in the signal and to measure the average uncertainty of the discrete signal. We used basic Shannon entropy developed by Shannon [23]. For given time series data $X=\left[\begin{array}{cccc} x_{1} & x_{2} & \cdots & x_{N} \end{array}\right]$, Shannon entropy value can be calculated by the following formula:

(3) $$\begin{array}{c} H=-\overset{k}{\underset{i=1}{\sum }}\left(\;p_{i}\;\right)\log_{2}\left(\;p_{i}\;\right), \end{array}$$

where *k* is the number of unique values in the data (*X*) and *p*_*i*_ is the probability (or normalized frequency) for these unique values.

### 3.6. Artificial Neural Network (ANN) Classifier

**Figure 6:**
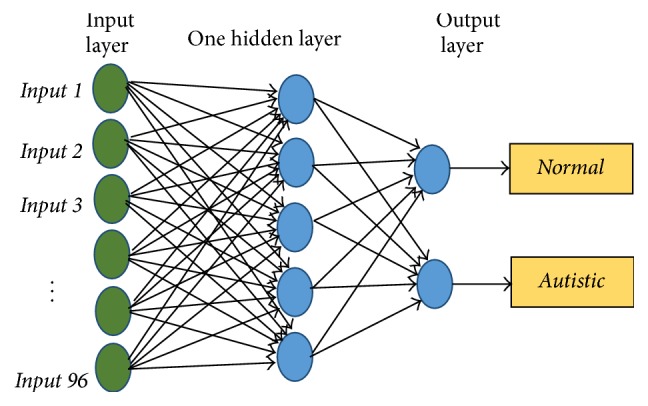
ANN structure.

ANN is widely used in biomedical engineering field such as modeling, data analysis, diagnostic, and detection. ANN is an information-processing system that is based on simulation of the human cognition process. ANN consisted of several computational neural units connected to each other. In this work, we design an ANN system with one input layer, one hidden layer, and one output layer.  Figure 6 shows our neural network structure. The hidden layer is designed with 5 nodes and log-sigmoid transfer function and output layer is designed with 2 nodes and soft-max (normalized exponential) transfer function.

The artificial neural network has to be trained to adjust the connection weights and biases in order to produce the desired mapping. At the training phase, the feature vectors are applied to the network which in turn adjusts its variable parameters, the weights, and biases, to capture the relationship between input patterns and outputs. The performance of ANN depends on the “epochs” process where epochs are the number of iterations of the training vectors used to update the weights of neurons.

### 3.7. Performance Evaluation

**Figure 7:**
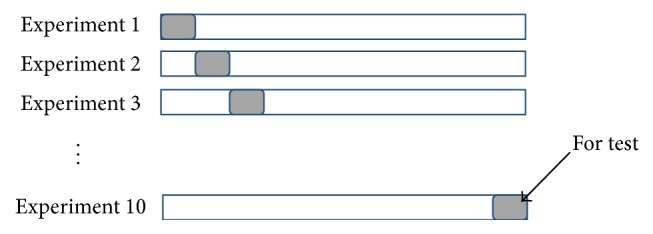
10-fold cross-validation procedures.

A well-known 10-fold cross-validation is used in all experiments. In the 10-fold cross-validation, the dataset is randomly divided into 10 equal parts (10 subsets). All the subsets are used for the training except one for the test (validation). This process is repeated 10 times (fold). Each subset is exactly used one time for testing data as shown in Figure 7. Thus, we ensure that all the examples in the features matrix are eventually used for both training and testing. The results of 10 times are averaged to produce a single classification performance.

In this current study we use the whole EEG recording for evaluation. The number of samples (or EEG segments) extracted from each subject depends on the segment length. Using one-minute (60 seconds) EEG segment length, we extracted 173 segments from autistic dataset and 148 segments from normal dataset. From these 321 segments, we select randomly 32 segments for testing and the remaining for training. As 10-fold cross-validation, this process is repeated 10 times and the results are averaged.

The performance is compared by considering* receiver operating characteristic* (ROC) parameters such as* true positive* (TP),* true negative* (TN),* false positive* (FP), and* false negative* (FN). True positive (TP) means that EEG segment from autistic subject ‎ is correctly diagnosed as autistic class. ROC graph shows the reliability of the classifier. The classification performance is evaluated in terms of sensitivity, specificity, and overall accuracy as in the following formulas:

(4) Sensitivity=TNFP+TN∗100,Specificity=TPTP+FN∗100,Accuracy=TP+TNTP+FP+TN+FN∗100.

The area under the ROC curve (AUC) is a common metric that can be used to compare different tests. An AUC is a measure of test accuracy. ROC curve describes two-dimensional visualization of ROC curve set for classifiers performance. The easiest possibility is to calculate the area under the ROC curve which is part of the area of the unit square. Consequently the value of AUC will always satisfy the following inequalities:

(5) $$\begin{array}{c} 0\leq AUC\leq 1. \end{array}$$

It is clear that if the AUC is close to 1 (area of unit square) AUC indicates very good test.

## 4. Results and Discussion

The experiments performed have two different scenarios. In the first one, the DWT output with statistical features (mean, standard deviation, etc.) directly as the input for the ANN classifier was used, while, in the second one, different entropy function in the DWT output, where the entropy values are then used as input for ANN classifier, was applied. After selecting the best feature extraction method, we performed some optimization techniques for further increasing of the accuracy. The experiments are carried out by using MATLAB 2013a software on windows 8 PC with Intel core i5 processor 2.30 MHz.

### 4.1. DWT with Statistical Features (without Entropy)

**Figure 8:**
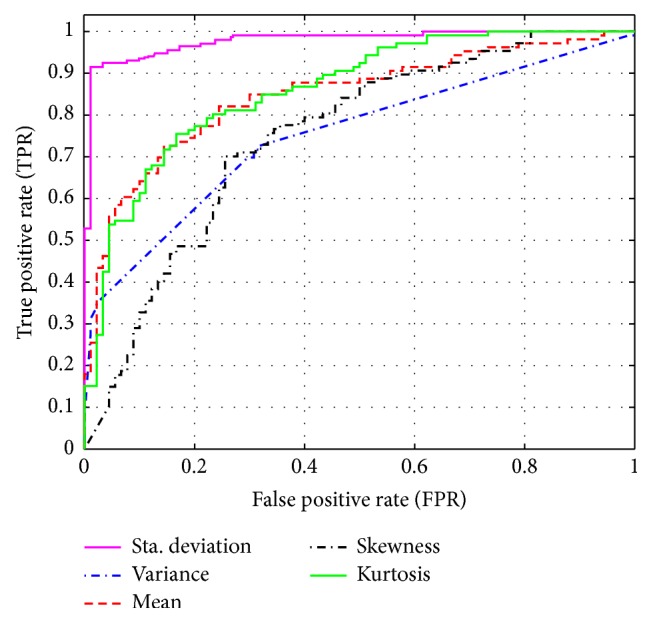
ROC curve with the statistical features.

In the first scenario, the discrete wavelet transform (DWT) technique and artificial neural network (ANN) classifier are used to detect the autistic signal without entropy function.  Table 2 summarizes the results and shows the classification accuracy rates for our approach, based on many statistical features such as mean, standard deviation, variance, skewness, and kurtosis.  Figure 8 shows the ROC curves for ANN classifier based on the previous statistical features. From Table 2 and Figure 8, it is clear that the classification accuracy is low. In the next step, we tried to apply several entropy functions to increase the accuracy.

### 4.2. DWT with Entropy Functions

**Figure 9:**
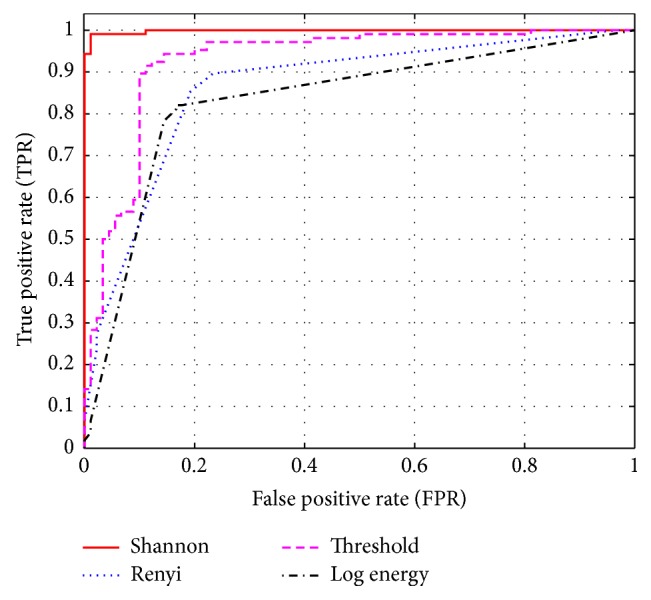
ROC curve with different entropy function.

In this scenario, DWT with different entropy functions to extract EEG feature were combined. We investigate four different entropy functions as described in the previous section: log energy and threshold entropies, Renyi entropy, and Shannon entropy.  Table 3 summarized the average classification accuracy according to the different types of entropies functions used. From Table 3 and Figure 9, it is clear that the best function to extract the features of an EEG signal is Shannon entropy.

### 4.3. Optimization Process

After selecting DWT + Shannon entropy as the best feature extraction method, we performed some optimization to further increase the accuracy. Optimization process is carried out by the following steps: (a) selecting the best segment length, (b) selecting best frequency band, and (c) testing between nonoverlapping and overlapping segments.

#### 4.3.1. Selecting the Best Segment Length

In the previous sections, the segment length is fixed to be ten seconds. In this section, we implemented our proposed approach with different window sizes (segment length).  Table 4 shows the classification accuracy obtained by our proposed approach on the 10-fold cross-validation method. The best result was achieved at segment length of 50 seconds with average accuracy up to 98.6%. We obtained lower accuracy using EEG segment shorter or longer than 50 seconds.

#### 4.3.2. Selecting Best Frequency Band (Wavelet Coefficients)

In this section, we investigated the effect of wavelet coefficients on the classification accuracy. In the previous sections, all five wavelet coefficients (D1–D4 and A4) and the original EEG segment are used for classification. In this section, we investigate the accuracy using different combination of wavelet coefficients.  Table 5 shows the summary of the results. It is clear that the best result was obtained using combination of all detail coefficients (D1 + D2 + D3 + D4).

#### 4.3.3. Testing the Effect of Nonoverlapping and Overlapping Segments

In this section, we studied the effect of overlapping segment on the classification accuracy. In (a), the all segments were nonoverlapping but in this section all the segments will be overlapped with half-segment, and window size at one minute and all detail bands (D1 + D2 + D3 + D4) were selected. By comparing Table 4 (nonoverlapping) and Table 6 (with overlapping), it is obvious that better results are obtained using overlapping EEG segment. The highest accuracy (99.8%) is obtained when the length of EEG segment is equal to 60 seconds with half-segment overlapping.

**Figure 10:**
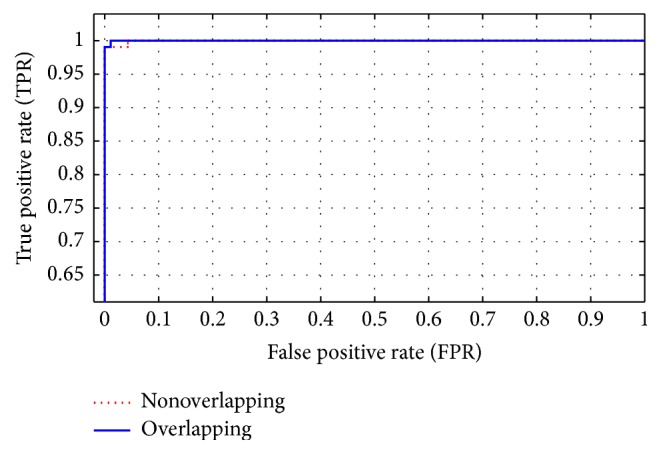
ROC curve for ANN classifier based on overlapping and nonoverlapping segments.

Figure 10 shows the ROC curves for ANN classifier when we used an entropy (En) function to improve the accuracy based on overlapping and nonoverlapping segments and one-minute (50 seconds) segment length. Therefore, Figure 10 shows that the area under ROC curve is almost one and the accuracy is close to the desired accuracy.

From the experimental results, we found that the good results are obtained from our approach when we used entropy function to get the features of an EEG signal. These results are obtained when the length of segment is one minute and when we choose the detail bands (D1 + D2 + D3 + D4) extracted by DWT and entropy function. All EEG signals are segmented by two ways: nonoverlapping and overlapping segments, but the best results are obtained at overlapping segments with accuracy up to 99.71%.  Table 7 summarized the best results. In the all previous results we did not employ ocular-artifact removal algorithm because it is very slow. However, when we employed the ocular-artifact removal algorithm, the accuracy is decreased up to 94%. This means that our artifact removal algorithm should be designed more properly and more investigation is needed.

Table 8 shows the comparison of our proposed method result with the existing methods. Detailed information about the existing methods has been presented in Section 2. It should be noted that most of proposed method is validated by different dataset that makes fair comparison for all methods slightly difficult. Tested using the same dataset, our method achieved higher accuracy (99.71%) than the method proposed by Alhaddad et al. [10] (90%). This shows the effectiveness of the proposed method for autism diagnosis. Another advantage of our proposed method is its simplicity. We use simple Shannon entropy that basically employs only arithmetic and log operations.

## 5. Conclusion 

A computer aided diagnosis (CAD) system has a tremendous potential to assist clinicians during the diagnosis process to save the time and increase the diagnosis accuracy. In this study, a CAD system was proposed in order to classify automatically autistic and nonautistic subject based on EEG signal analysis. Firstly, only discrete wavelet transform (DWT) with statistical features (mean, standard deviation, variance, skewness, and kurtosis) was employed to extract the features of EEG signal. The artificial neural network ANN classifier is used to classify the subject based on the extracted features. However with only DWT and statistical features for feature extraction the classification accuracy is low. We then investigated several entropy functions for feature extraction: log energy and threshold entropies, Renyi entropy, and Shannon entropy.

The highest classification accuracy is obtained with combination of DWT and Shannon entropy for feature extraction. After some optimization process we obtained classification accuracy up to 99.71%. The processing time for feature extraction and classification was also fast enough due to the simplicity of the proposed method. Further research will include testing the proposed method using larger dataset. Adaptive learning to improve the CAD system performance over the time will be investigated as well. We consider also doing more investigation on preprocessing step, especially in eye-artifact removal, in our future work.

## Figures and Tables

**Table 1:** Frequency bands for each wavelet coefficient.

Wavelet coefficients	EEG subbands	Frequency (Hz)
D1	—	128–256
D2	—	64–128
D3	Gamma	32–64
D4	Beta	16–32
A4	Alpha, theta, delta	0–16

**Table 2:** The classification accuracy rates with the statistical features.

Statistical features	Accuracy (%)
Mean	78
Standard deviation	96
Variance	70
Skewness	70
Kurtosis	79

**Table 3:** Classification accuracy with different entropy functions.

Entropy functions	Accuracy (%)
Log energy	83
Threshold	88.6
Renyi entropy	83.2
Shannon entropy	98.4

**Table 4:** The average classification accuracy for different segments lengths.

Segment length (*S*)	Accuracy (%)
10	96.8
20	97.2
30	97.5
40	98.0
*50*	*98.6*
60	98.1
90	97.6
120	95.1
150	93.7
180	91.6

**Table 5:** The effect of combination of wavelet coefficient on the classification performance.

Combination of wavelet coefficients (freq. band)	Accuracy (%)
D2 (64–128 Hz)	94.8
D3 (32–64 Hz)	94.8
D4 (16–32 Hz)	94.6
A4 (0–16 Hz)	88
Original EEG + D1 + D2 + D3 + D4 + A4	98.6
D1 + D2 + D3 + D4 + A4	98.4
D2 + D3 + D4 + A4	97.3
*D1 + D2 + D3 + D4*	*98.9*
D1 + D2 + D3	97.8
D2 + D3 + D4	96.7
D2 + D3	95.8
D1 + D2	92.7

**Table 6:** The classification accuracy with overlapping (half-segment).

Segment length (*S*)	Accuracy (%)
10	98.4
20	98.6
30	98.7
40	99.4
50	99.7
60	99.6
90	99.5
120	99.3
150	99.3
180	99.1

**Table 7:** Summary of final results of the proposed method.

Segments	Frequency bands	Length of segment	Feature extraction	Classifier	Cross-validation method	Classification accuracy average
Nonoverlapping	D1 + D2 + D3 + D4	50 seconds	DWT-En	ANN	10-fold	98.6%
Overlapping (with half-segment)						99.7%

**Table 8:** Several EEG-based CAD of autism spectrum disorder.

Author	Feature extraction	Classifier	Dataset	Acc (%)
Sheikhani et al. 2008 [4]	STFT, coherence	KNN	Own dataset	82.4
Ahmadlou et al. 2010 [6]	Wavelet, fractal dimension (FD)	RBNN	Own (Iranian dataset)	90
Bosl et al. 2011 [9]	Modified multiscale entropy (MMSE)	SVM	Own (USA)	70–100
Sheikhani et al. 2012 [5]	STFT, coherence	KNN	Own dataset	96.4
Ahmadlou et al. 2012 [7]	Wavelet, visibility graph (VG)	EPNN	Iranian dataset	95.5
Ahmadlou et al. 2012 [8]	Wavelet, fuzzy SL	EPNN	Iranian dataset	95.5
Alhaddad et al. 2012 [10]	FFT	FLDA	Own (KSA dataset)	90
Our work	DWT, Shannon entropy	ANN	KSA dataset	99.7
